# 

**Published:** 1897-06

**Authors:** 


ESTABLISHED 1855. SUBSCRIPTION, $2.00.

### ATLANTA MEDICAL AND SURGICAL JOURNAL.

VOLUME XIV NEW SERIES. ATLANTA, GA., JUNE, 1897. No. 4.

